# Supplementary material for: Independent evaluation of Wolbachia infected male mosquito releases for control of Aedes aegypti in Harris County, Texas, using a Bayesian abundance estimator
Source: PLoS Negl Trop Dis. 2022 Nov 14;16(11):e0010907. doi: 10.1371/journal.pntd.0010907 (PMC9704758; doi:10.1371/journal.pntd.0010907)
Supplement: S1 File — (DOCX) [file pntd.0010907.s001.docx]

### *Spatial Aggregation: Introduction*

An unmodified Poisson process is usually a starting point for modelling ecological count data. However, ecological count data commonly shows greater variation than the expected values of a Poisson distribution. In our case, mosquito count data were expected to be somewhat overdispersed. To assess mosquito spatial aggregation ***λ*** was described with a gamma distribution [1-5] and as a linear regression as follows:

$\boldsymbol{\lambda} \sim\mathrm{Gamma}\left( \boldsymbol{\alpha}, \boldsymbol{\beta} \right)$.

Later the aggregation was estimated using Taylor’s equation 2.6a [2] ($\boldsymbol{r} \sim\frac{\boldsymbol{\mu}^{2}}{\boldsymbol{\sigma}^{\boldsymbol{2}}- \boldsymbol{\mu}}$), where $\boldsymbol{\mu}= \frac{\boldsymbol{\alpha}}{\boldsymbol{\beta}}$ and $\boldsymbol{\sigma}^{\boldsymbol{2}}\boldsymbol{=}\frac{\boldsymbol{\beta}+1}{\boldsymbol{\beta}}\boldsymbol{\cdot}\frac{\boldsymbol{\alpha}}{\boldsymbol{\beta}}$ [6]. With this approach, we are effectively saying that, after accounting for the trapping error, the population process followed a gamma-Poisson distribution. The advantage of this parametrization is that it accounts for specimen aggregation (a.k.a., overdispersion, clustering, heterogeneity, etc.) while preserving the estimate of the mean of the population [3, 7]. The dispersion index (***DI***) was calculated as the reciprocal of the ***r*** parameter (1/***r***) [2, 3] (commonly expressed as 1/***k*** in the ecological literature). If ***DI*** is > 0 the population is considered aggregated, and if ***DI*** = 0 the population is considered randomly distributed. However, this commonly used index has limitations and is heavily correlated with the abundance of the specimens under study [4].

### *Spatial Aggregation: Results*

The mosquito population for both species, in both areas, and in all weeks showed indications of spatial aggregation with a ***DI*** statistically greater than zero (Supp 1. Table 1). Though *Ae. aegypti’s* *DI* in the treatment area (TA) was not statistically different at any time, it showed considerable increases in uncertainty after week 28; the lowest observed ***DI*** was in week 28 (***DI*** = 0.06 (0.01, 1.84)) and the highest in week 33 (***DI*** = 1.15 (0.08, 36.01)). Paradoxically, given the large observed uncertainty, we cannot state that the *Ae. aegypti* TA population became more aggregated because of the releases. Our results show known difficulties of estimating spatial aggregation when the number of specimens is very low because 1/***r*** behaves erratically at low specimen densities [47].

Supp. Table 1. Dispersion index (*DI*) by week for *Ae. aegypti* and *Ae. albopictus* females in the UA and TA.

|  | Untreated Area | | Treatment Area | |
| --- | --- | --- | --- | --- |
| W | *Ae. aegypti* | *Ae. albopictus* | *Ae. aegypti* | *Ae. albopictus* |
| 28 | 0.05 (0.01, 1.67) | 0.10 (0.01, 3.07) | 0.06 (0.01, 1.84) | 0.06 (0.01, 1.79) |
| 29 | 0.07 (0.01, 2.09) | 0.06 (0.01, 2.01) | 0.13 (0.01, 4.20) | 0.05 (0.01, 1.34) |
| 30 | 0.05 (0.01, 1.49) | 0.04 (0.01, 1.24) | 0.24 (0.02, 8.21) | 0.04 (0.005, 1.10) |
| 31 | 0.06 (0.01, 1.73) | 0.06 (0.01, 1.94) | 0.44 (0.03, 14.25) | 0.06 (0.01, 1.78) |
| 32 | 0.06 (0.01, 1.80) | 0.05 (0.01, 1.58) | 0.44 (0.03, 14.74) | 0.04 (0.01, 1.19) |
| 33 | 0.06 (0.01, 1.64) | 0.04 (0.01, 1.21) | 1.15 (0.08, 36.01) | 0.03 (0.004, 0.94) |
| 34 | 0.06 (0.01, 1.86) | 0.07 (0.01, 2.10) | 0.56 (0.03, 18.78) | 0.04 (0.01, 1.26) |
| 35 | 0.04 (0.01, 1.27) | 0.09 (0.01, 2.92) | 0.79 (0.04, 25.70) | 0.03 (0.01, 0.92) |
| 36 | 0.05 (0.01, 1.68) | 0.03 (0.01, 0.88) | 0.90 (0.05, 28.34) | 0.04 (0.01, 1.11) |
| 37 | 0.08 (0.01, 2.76) | 0.08 (0.01, 2.68) | 0.46 (0.03, 15.05) | 0.06 (0.01, 1.60) |
| 38 | 0.25 (0.02, 8.41) | 0.32 (0.02, 10.56) | 0.81 (0.04, 26.52) | 0.22 (0.01, 7.45) |

W = week of the year; Values are Dispersion Indices with their corresponding 95% credibility intervals inside parentheses.

Cited Literature

1. Johnson NL, Kemp AW, Kotz S. Univariate Discrete Distributions: Wiley; 2005.

2. Taylor RAJ. Chapter 2 - Spatial pattern. In: Taylor RAJ, editor. Taylor's Power Law: Academic Press; 2019. p. 13-25.

3. Lindén A, Mäntyniemi S. Using the negative binomial distribution to model overdispersion in ecological count data. ECOLOGY. 2011;92(7):1414-21. doi: 10.1890/10-1831.1.

4. Taylor LR, Woiwod IP, Perry JN. The Negative Binomial as a Dynamic Ecological Model for Aggregation, and the Density Dependence of k. Journal of Animal Ecology. 1979;48(1):289-304. doi: 10.2307/4114.

5. Gelman A, Carlin JB, Stern HS, Dunson DB, Vehtari A, Rubin DB. Bayesian data analysis. Bayesian Data Analysis. Texts in Statistical Science Series. Third Edition ed. Boca Raton, FL: Chapman and Hall; 2013. p. 141-62.

6. Gelman A, Carlin JB, Stern HS, Dunson DB, Vehtari A, Rubin DB. Models for robust inference. Bayesian Data Analysis. Texts in Statistical Science Series. Third Edition ed. Boca Raton, FL: Chapman and Hall; 2013. p. 435-47.

7. Holmes S, Huber W. Mixture Models. Modern statistics for modern biology. First ed. New York, US: Cambridge University Press; 2018. p. 83-106.
