# Supplementary figures and images for: Independent evaluation of Wolbachia infected male mosquito releases for control of Aedes aegypti in Harris County, Texas, using a Bayesian abundance estimator
Source: PLoS Negl Trop Dis. 2022 Nov 14;16(11):e0010907. doi: 10.1371/journal.pntd.0010907 (PMC9704758; doi:10.1371/journal.pntd.0010907)

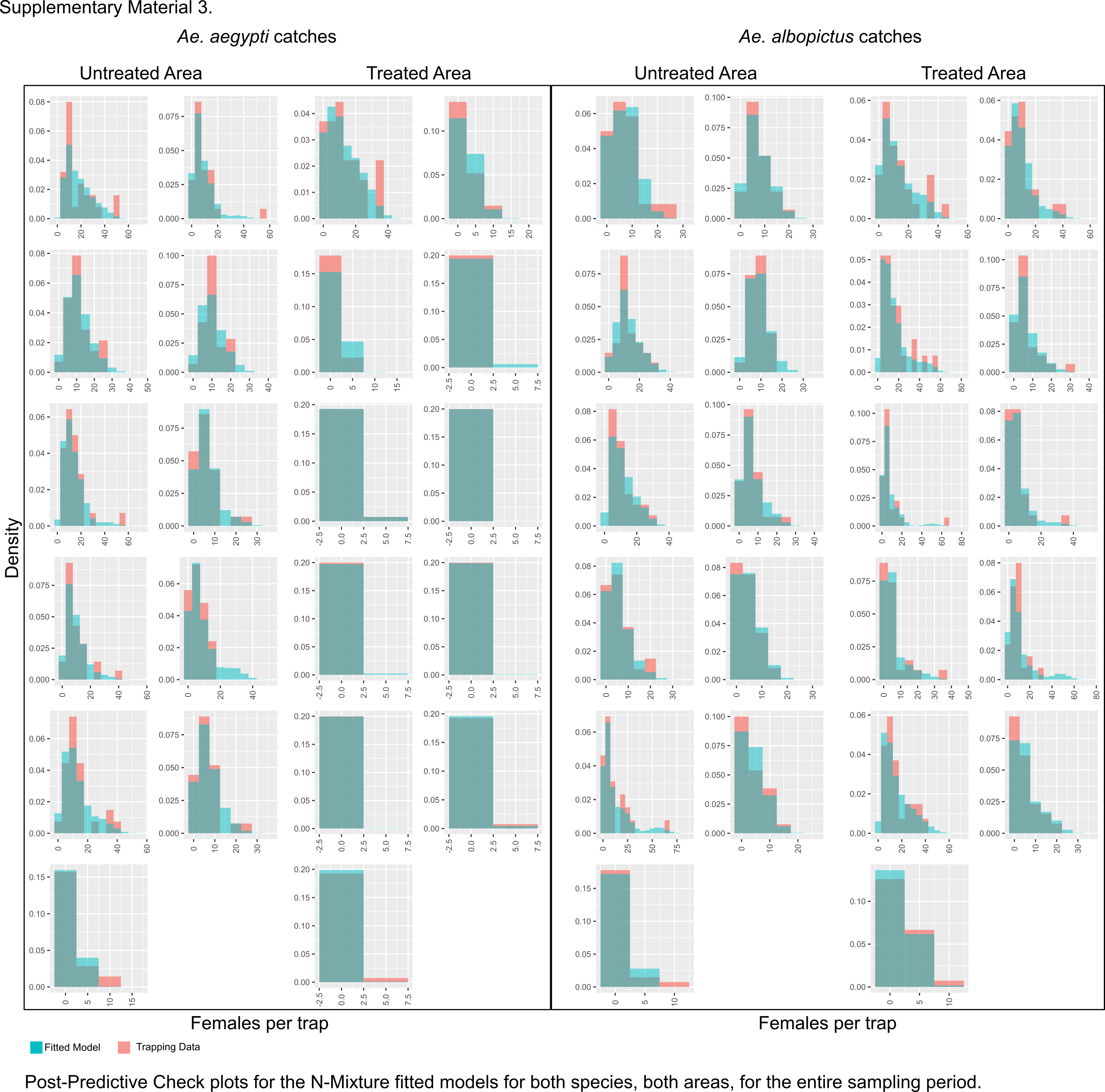

Supplement: S1 Fig — Blue histograms are the best fitted model; Red histograms are the trapping data. A properly fitted model will cover most of the trapping data histogram. (TIF) [file pntd.0010907.s004.tif]
