# Supplementary material for: Independent evaluation of Wolbachia infected male mosquito releases for control of Aedes aegypti in Harris County, Texas, using a Bayesian abundance estimator
Source: PLoS Negl Trop Dis. 2022 Nov 14;16(11):e0010907. doi: 10.1371/journal.pntd.0010907 (PMC9704758; doi:10.1371/journal.pntd.0010907)
Supplement: Attachment — Submitted filename: replies_to_reviewer_3.docx [file pntd.0010907.s005.docx]

Reviewer's Responses to Questions

Key Review Criteria Required for Acceptance?
As you describe the new analyses required for acceptance, please consider the following:

Methods
-Are the objectives of the study clearly articulated with a clear testable hypothesis stated?
-Is the study design appropriate to address the stated objectives?
-Is the population clearly described and appropriate for the hypothesis being tested?
-Is the sample size sufficient to ensure adequate power to address the hypothesis being tested?
-Were correct statistical analysis used to support conclusions?
-Are there concerns about ethical or regulatory requirements being met?

Reviewer #3: The study design was clear and appropriate to accomplish the objectives of the study, with treatment and control sites chosen for release and regular mosquito sampling. The study was conducted in Texas, which has an abundant Ae. aegypti population with demonstrated spread of Zika. It seems a reasonable location to test new control measures, particularly given the potential for compensatory increases in Ae. albopictus, which might be more concerning in an area where DENV is endemic.

My primary concern with the Methods – and the paper as a whole – is that the analyses conducted do not integrate the important covariates. The primary objective of comparing mosquito abundance between the treated and untreated locations is done by comparing the mosquito abundance estimates generated from separate N-mixture models. What’s more, this is done on a week-by-week basis, meaning that all statements regarding trends and temporal sequences are based on eyeballing graphs, not on statistical inference. Even if the N-mixture model is so limited that it can only estimate the mosquito abundance and its 95% CI, the negative binomial should have been able to incorporate terms for treatment and week. This becomes especially important for the analyses of Ae. albopictus, which seem to rely entirely on eyeballing the correspondence between the climatic graphs and the Ae. albopictus graphs/estimates.

We will address each concern raised in the following paragraphs.

*Treatment (or time) were not incorporated as covariables, not because of the inability of the N-mixture model, but because we think we did not have enough degrees of freedom to spend. Consider that there were no replications. The reduction signal was clear, we thought that by adding parameters to the model it would only add complexity to the interpretation of the model (and the results), increase the risk of overfitting and model selection bias. Additionally, from experience we always run into push-back from reviewers when presenting “complicated”, “highly mathematical”, “non-biological” models (see comments by reviewer #2 for an example).

*The analysis was, in fact, not done week-by-week. We did not include the temporal subscript, however, notice that temporal grouping is present in the code (supplemental materials); we added the subscript to the model’s algebra and described it verbally.

*Regarding the having separate N-mixture models. We ran again the analysis in the “same N-mixture model” and obtained equivalent results to the ones presented; however, we removed the dispersion index parameters. We added a linear regression component to the population process, where treatment was a covariable (0 for untreated, 1 for treated). Here is where things get a little complicated because there are several options on how to treat the intercept and slope of the linear regression (something that is commonly obscured in biological publications; see Bayesian Hierarchical Models in Ecology by Steve Midway for a discussion on this). Because we have no information or can put forward an informed guess, we assumed that each week had a different regression, i.e., the effect of the treatment was allowed to not be the same week-to-week, the slope and intercept are flexible. The following is the algebra of the new model where the subscript **i** denotes the trap, **j** the repeated sampling, and **k** the week.

trapping process:

$\boldsymbol{C}_{\boldsymbol{ikj}}\boldsymbol{|}\boldsymbol{N}_{\boldsymbol{i}\boldsymbol{k}}\boldsymbol{\sim}\mathrm{Binomial}\left( \boldsymbol{\tau}_{\boldsymbol{ik}}\boldsymbol{,}\boldsymbol{N}_{\boldsymbol{ik}} \right)$;

population state process:

$$\boldsymbol{N}_{\boldsymbol{ik}}\boldsymbol{\sim}\mathrm{Poisson}\left( \boldsymbol{\lambda}_{\boldsymbol{ik}} \right)$$

$$\log\left( \boldsymbol{\lambda}_{\boldsymbol{ik}} \right)={\boldsymbol{\beta}_{\boldsymbol{0}}}_{\boldsymbol{ik}}+{\boldsymbol{\beta}_{\boldsymbol{1}}}_{\boldsymbol{ik}}\boldsymbol{\cdot}\boldsymbol{treatment}_{\boldsymbol{ik}}$$

The following are the simulation results:

Saved parameters: lambda totalMosquitoes mosquitoes te beta0 beta1 deviance

MCMC ran in parallel for 4.691 minutes at time 2021-12-09 11:57:54.

For each of 4 chains:

Adaptation: 100 iterations (sufficient)

Burn-in: 20000 iterations

Thin rate: 1 iterations

Total chain length: 50100 iterations

Posterior sample size: 30000 draws

Successful convergence based on Rhat values (all < 1.1).

DIC info: (pD = var(deviance)/2)

pD = 627.5 and DIC = 4590.404

Because this a different single model we cannot use the DIC to compare fits between this and the submitted models, however, the Post-Predictive Checks are almost identical to the submitted (let us know if you would like to see the plots). In Fig 1 (below) we show that the results are statistically identical, except for week 29 where the abundance between the untreated and the treated areas are no longer statistically different. Also, notice that error bars are now a little larger in the treated area results.

Fig 1. Ae. aegypti relative abundance. The center dot denotes the most probable value of the estimate. Error bars denote 95% credibility intervals. Treatment covariate.

We also ran the model with **treatment** and the **week (1-11)** as covariates, such that:

trapping process: $\boldsymbol{C}_{\boldsymbol{ijk}}\boldsymbol{|}\boldsymbol{N}_{\boldsymbol{ik}}\boldsymbol{\sim}\mathrm{Binomial}\left( \boldsymbol{\tau}_{\boldsymbol{ik}}\boldsymbol{,}\boldsymbol{N}_{\boldsymbol{ik}} \right)$;

population state process:$\boldsymbol{N}_{\boldsymbol{ik}}\boldsymbol{\sim}\mathrm{Poisson}\left( \boldsymbol{\lambda}_{\boldsymbol{ik}} \right)$

$\log\left( \boldsymbol{\lambda}_{\boldsymbol{ik}} \right)={\boldsymbol{\beta}_{\boldsymbol{0}}}_{\boldsymbol{ik}}+{\boldsymbol{\beta}_{\boldsymbol{1}}}_{\boldsymbol{ik}}\boldsymbol{\cdot}\boldsymbol{treatment}_{\boldsymbol{ik}}\boldsymbol{+}{\boldsymbol{\beta}_{\boldsymbol{2}}}_{\boldsymbol{ik}}\boldsymbol{\cdot}\boldsymbol{week}_{\boldsymbol{ik}}$.

The model fitting was successful.

Saved parameters: lambda totalMosquitoes mosquitoes te beta0 beta1 beta2 deviance

MCMC ran in parallel for 4.786 minutes at time 2021-12-10 12:09:09.

For each of 4 chains:

Adaptation: 100 iterations (sufficient)

Burn-in: 20000 iterations

Thin rate: 1 iterations

Total chain length: 50100 iterations

Posterior sample size: 30000 draws

Successful convergence based on Rhat values (all < 1.1).

DIC info: (pD = var(deviance)/2)

pD = 634.3 and DIC = 4597.66

The results were statistically identical to the previous model (Fig 2) because **week** did not have an effect on **λ** (Table 1), while **treatment** was statically different from zero (i.e., zero is not contained the 95% credibility intervals) validating a detectable effect after week 28. The **intercept** was not different from zero for any of the weeks, and the overall fit of the model to the data was not improved by the addition of the **week** covariate.

Fig 2. Ae. aegypti relative abundance. The center dot denotes the most probable value of the estimate. Error bars denote 95% credibility intervals. Treatment and Week covariates.

Table 1. Values for β1 and β2.

| Week | β_1_ | β_2_ |
| --- | --- | --- |
| 28 | 0.17 (-0.13, 0.47) | 1.36 (-137.32, 140.77) |
| 29 | 0.79 (0.39, 1.19) | 0.89 (-86.78, 88.69) |
| 30 | 2.30 (1.87, 2.74) | 0.31 (-61.69, 62.38) |
| 31 | 3.14 (2.62, 3.67) | 0.02 (-47.58, 47.79) |
| 32 | 2.97 (2.48, 3.48) | 0.14 (-38.19, 38.74) |
| 33 | 4.66 (3.87, 5.65) | -0.26 (-32.56, 31.85) |
| 34 | 3.32 (2.77, 3.91) | -0.01 (-27.74, 27.84) |
| 35 | 4.46 (3.77, 5.21) | -0.09 (-24.54, 24.20) |
| 36 | 4.10 (3.46, 4.85) | -0.04 (-21.70, 21.53) |
| 37 | 2.36 (1.87, 2.86) | 0.07 (-19.42, 19.64) |
| 38 | 1.77 (1.00, 2.62) | -0.04 (-17.75, 17.65) |

We would be happy to submit any of the three modelling approaches. However, we will need help to persuade some reviewers to allow the proper methodological description.

*We agree that Ae. albopictus is based on interpretation and expert opinion. We did not make any statistical statements regarding the effect of weather and mosquito abundance. Again, the experiment had no replication, and was not designed to control for weather. We added to the manuscript the climatic and weather data to provide a larger picture of what could had influence the mosquito abundance in addition to the treatment.

Results
-Does the analysis presented match the analysis plan?

-Are the results clearly and completely presented?

-Are the figures (Tables, Images) of sufficient quality for clarity?

Reviewer #3: Several of the figures, particularly 1, 4 and 6, brought significant clarity to the Results. The order of the Results text was confusing, though, with ‘Experimental Results’ first, but then the abundance results actually not presented in that section, despite those being the entire point of the experiment.

We have removed the section title ‘Experimental Results’ and added the sentences “The paragraphs that follow provide a general description of the trapping area, the total number of female mosquitos caught (by species and area), and the model fit results. Following that, we present the N-mixture model's predictive power as well as results for Ae. aegypti and Ae. albopictus abundance.”

My only other major comment about the Results regards the text, “Notwithstanding the PPC results” (line 260), which I don’t see discussed anywhere. The figure in the Supplement is difficult to interpret. Is there something concerning here that calls the other results into question?

We have change, the sentence. There is nothing concerning about the fits; we used the adjective incorrectly trying to say that the same results were observed in the numeral approach (DICs). The supplemental figure was cut by the editorial manager showing only one species and one statistical approach.

--------------------

Conclusions
-Are the conclusions supported by the data presented?

-Are the limitations of analysis clearly described?

-Do the authors discuss how these data can be helpful to advance our understanding of the topic under study?

-Is public health relevance addressed?

Reviewer #3: I find the main conclusion – that the intervention worked – generally compelling despite the limitations of the modeling noted above. The differences shown in Figure 4 are so large, that a more efficient modeling approach would do little to change the conclusion, I imagine. However, I am not at all convinced by the conclusions about the effects on Ae. albopictus. Those results (Figure 6) are too close, and the explanations about climatologic variables need statistical analysis to back them up. Compensatory increases in Ae. albopictus are a serious side effect and have to be critically considered.

We have addressed the modelling issues; we agree that an approach with more statistically power was unlikely to change the conclusion about the intervention; we also show that to be the case with the inclusion of **treatment** and **week** as covariates. Regarding Ae. albopictus, the experimental design does not control for weather, and from experience we know the sampling was relatively short to attempt adding weather parameters, for these reasons we are using expert opinion, but we are also providing the raw data should anyone attempt to model the population dynamics using weather.

Editorial and Data Presentation Modifications?
Use this section for editorial suggestions as well as relatively minor modifications of existing data that would enhance clarity. If the only modifications needed are minor and/or editorial, you may wish to recommend “Minor Revision” or “Accept”.

Reviewer #3:

1. The full presentation of average number of mosquitoes per trap and total number per hectare-0.1, and inclusion of a figure for both, feels unnecessary, given that they show the same results.

Tough they showed different information — one was the average number of females, the other was the total number of mosquitoes — the number of traps varied little and as consequence the patterns are the same. Regardless, we removed the figure showing total of Ae. aegypti females.

2.      I wasn’t aware of this issue before reading this paper, and so had to go do some research on it, but it appears that overdispersion, a distribution with higher than expected variance, has apparently been redefined to some extent in the ecological literature to be conflated with aggregation, the clustering together of population subsets. That seems to be the definition used here, as implied on lines 218-219. However, the kind of overdispersion handled by a negative binomial model is the statistical kind. I am only familiar with it in the Poisson/negative binomial context and cannot judge the appropriateness of this conflation with aggregation in the N-mixture model. I will note, however, that the spatial aggregation results (page 22) don’t really enter into the Discussion/Conclusions in any meaningful way, so the emphasis it receives doesn’t seem justified at present.

Agreed. This section was added because the issue of using Poisson vs a Negative Binomial distribution was heavily brought up in a previous review, but without getting into ecological/statistical theory the discussion of the dispersion index results carries no weight; such discussion has been disliked by reviewers. Statistical overdispersion (and underdispersion) typically results when the structure in the mean response is ignored and not accounted with the “correct” covariates — e.g., clustering of individuals in groups, or spatial (or temporal) correlation. As you mentioned, some ecological literature has conflated statistical overdispersion with the aggregation of individuals in space, which indeed can lead to statistical overdispersion but is not the only mechanism. Furthermore, using the negative binomial in place of the Poisson distribution will not always solve issues of statistical overdispersion; not accounting for overdispersion can generate optimistic results because the standard errors and confidence interval will be too short. We took an extremely cautious approach by accounting for statistical dispersion using a direct measurement (i.e., not just dividing variance/mean). We can remove this measurement saving degrees of freedom and move the current analysis to supplemental material.

3.      Lines 96-97. I am not sure what the data from FL is supposed to tell me here.

The sentence has been changed; we removed the FL data.

4.      Lines 115-116. On what basis were the sites selected?

Based on their housing similarity and location within Harris County 4^th^ precinct.

5.      Line 117. Can you relate this distance to a mosquito’s range to contextualize it for readers?

We have added two references on mosquito dispersal.

6.      Lines 167-174. Are the parentheses in the correct places here? Order of operations would dictate that TA/UA is multiplied by 100 FIRST, before it’s subtracted from 1. The percent reduction would be (1-(TA/UA))x100.
Agreed, there parentheses were not placed correctly; the presented results used the correct order of operation. We have made the correction in the text.

7.      Line 272. Is there a reason all 5 weren’t used? Does the method work equally well in the other two?

The other two colors had not been analyzed; to Dr. Lozano’s recollection the colors were picked at random.

In the other two experiments, Villela et al. recovered 30 green marked females and 35 yellow marked females. For green, 20 separate MCMC simulations failed to converge (rhat > 1.1) , even with very large runs (> one hr.). For yellow the model returned a N = 454[ 58, 2466]. In our opinion the model continues to perform well, however there might be a sample size issue with the green releases. We have changed the paragraph to include these results.

8.      Lines 274-277. All were underestimates with large confidence intervals. This wasn’t discussed in relation to the study results. How would you anticipate this could be impacting the results?

Regarding the underestimation, our approach was off by less than 20% of the true value (without any optimizations) for the 4 MMR experiments we now present. Does that mean that it will be off for the next dataset? Is this a common feature of this modelling approach? Of course, this is unknow because we do not have access to more MMR datasets to ascertain it.

To tackle this issue, we used a very cautious approach to estimating the reduction’s intervals by comparing the upper and lower limits and not only the most probable values like it is commonly done. If measurements for a particular week had large uncertainty around the abundance, the reduction’s uncertainty will also grow considerably. Notice the intervals for week 29 with a reduction of 55% and an uncertainty of 24 and 73%. Just for clarification, confidence intervals provided by frequency base statistics (i.e., Fisher and MLE statistics) are not probabilistic, while credibility interval obtained from Bayesian statistics are indeed probabilistic.

We added a summary of these paragraphs to the discussion section.

9.      Line 309. What are these referring to?

This section has been removed. But for clarification, the relative abundance, **λ,** is the average number of females per **i** trap, while **N_i_** (a latent variable) is the total number of females in the area of influence of trap **i**. Therefore, the total number of females in the trapping area is the sum of all **N_i_**, or **λ** times the number of traps.
10.     Line 338. Why is the minimum temperature here (18.9) different than the min. of the three days it’s pulled from (line 337)? Typo?

19.8 °C was the weekly minimum average, while 18.9 °C was the minimum temperature for July 24th.
--------------------

Summary and General Comments
Use  this section to provide overall comments, discuss strengths/weaknesses of the study, novelty, significance, general execution and scholarship. You may also include additional comments for the author, including concerns about dual publication, research ethics, or publication ethics. If requesting major revision, please articulate the new experiments that are needed.

Reviewer #3: Lozano et al. performed an independent investigation of a commercial Ae. aegypti control intervention that avoids building concerns regarding insecticide resistance using an Incompatible Insect Technique. Leveraging treated and untreated areas with Ae. aegypti released by the manufacturer, the investigators collected mosquitoes at regular intervals. They used a novel statistical technique to account for underestimates of mosquitoes in traps and ultimately showed drastic decreases in Ae. aegypti in the treated zone. They argue that apparent increases in Ae. albopictus in the treated area are due to climatic variation and not due to the intervention. Overall, the study was well-conducted and compellingly shows that that the Incompatible Insect Technique is successful in reducing Ae. aegypti abundance.

However, limitations of the statistical methods used leave open the question of whether the Ae. albopictus population responded with increased abundance, which is an important public health consideration.

We agree there are limitations in the Aedes aegypti / Aedes albopictus statistical design; consider the number of replications and temporal sampling (11 weeks) which were not under our control. The language we use when presenting the Ae. albopictus results are non-statistical and clearly not emphatic. We added another sentence to deemphasize this result.
