# Supplementary material for: Independent evaluation of Wolbachia infected male mosquito releases for control of Aedes aegypti in Harris County, Texas, using a Bayesian abundance estimator
Source: PLoS Negl Trop Dis. 2022 Nov 14;16(11):e0010907. doi: 10.1371/journal.pntd.0010907 (PMC9704758; doi:10.1371/journal.pntd.0010907)
Supplement: Attachment — Submitted filename: replies_to_review_of_v2.docx [file pntd.0010907.s006.docx]

Dear Lozano,

Thank you very much for submitting your manuscript "Independent evaluation of Wolbachia infected male mosquito releases for control of Aedes aegypti in Harris County, Texas, using a Bayesian abundance estimator" for consideration at PLOS Neglected Tropical Diseases. As with all papers reviewed by the journal, your manuscript was reviewed by members of the editorial board and by several independent reviewers. The reviewers appreciated the attention to an important topic. Based on the reviews, we are likely to accept this manuscript for publication, providing that you modify the manuscript according to the review recommendations.

Please prepare and submit your revised manuscript within 30 days. If you anticipate any delay, please let us know the expected resubmission date by replying to this email.

When you are ready to resubmit, please upload the following:

[1] A letter containing a detailed list of your responses to all review comments, and a description of the changes you have made in the manuscript.

Please note while forming your response, if your article is accepted, you may have the opportunity to make the peer review history publicly available. The record will include editor decision letters (with reviews) and your responses to reviewer comments. If eligible, we will contact you to opt in or out

[2] Two versions of the revised manuscript: one with either highlights or tracked changes denoting where the text has been changed; the other a clean version (uploaded as the manuscript file).

Important additional instructions are given below your reviewer comments.

Thank you again for your submission to our journal. We hope that our editorial process has been constructive so far, and we welcome your feedback at any time. Please don't hesitate to contact us if you have any questions or comments.

Sincerely,

Amy C Morrison, PhD

Section Editor

PLOS Neglected Tropical Diseases

Amy Morrison

Section Editor

PLOS Neglected Tropical Diseases

There was consensus among the editorial board that this manuscript should have a path to publication, and ask that your revised manuscript carefully address the remaining comments from Reviewer #3. In addition, below are some observations from the Section Editor that I suggest the authors consider. No need to provide a point by point response to the editor comments, but hope they may influence the next revision.

1. The manuscript presents data from an independent evaluation if a Wolbachia-based population suppression strategy in the city of Houston, TX. From this data, there is compelling evidence that the strategy was effective during the period of evaluation, even though the study methodology had numerous limitations, including but not limited to small sample size (only 26-28 BG traps each in an untreated an treated area, no replicates (one treated and untreated area) and for me no significant comparison of the two areas prior to implementing the intervention.

Agreed. We discuss these limitations.

2. The authors use a relatively novel statistical approach to analyze the data which seems reasonable to me. It is clear the authors have a strong grasp of the statistics and are able to discuss the advantages and disadvantages over other methods such as negative bionomial regression. As far as I can discern the trap data is pretty convincing anyway you do the analysis. The argument that the their method accounts for trapping biases was not all that convincing to me especially since they used a single mark release capture study to "parameterize" (this was my non-statistical interpretation).

Without getting into details, we did not use the results of the MMR experiments information because we, the mosquito community, do not have experience on how to include prior information in a new estimation (using data from a previous experiment to help the parameter estimation in a new experiment.) We used the MRR experiment data to give us confidence that it is possible to recover the “true” number of specimens in an area with only trapping data.

Although it is simple to add prior information in Bayesian inference, how many in our community would be able to verify that the addition is appropriate (sound parameter ranges, distributions, etc.)? Thus, we used what are called non-informed priors, i.e., we did not use external information, which give the same results of an MLE estimation. Just like in MLE, Bayesian inference explores the parameter space and finds (hopefully) the best values to explain the data, however there are fundamental differences between Bayesian inference and MLE [1].

3. Those of us who sample Aedes aegypti, no that there are always a lot of houses with zero trap totals (or really don't have Ae. aegypti), work I've don't personally shows that distributions don't have much spatial structure beyond an individuals household, but the distribution of infested houses change overtime. Our conclusion has always been you need large sample sizes. Again this study was not outstanding in size but large enough in my view to measure the impact of the intervention.

We agree with some of these observations, we moved the adult aggregation metric (that can be produce by data overdispersion) to supplemental materials.

We somewhat disagree with the observation about sample size because “how large is large?” During sample size estimation for abundance reduction, one should consider finding the “true” parameters of two populations and the size of the difference one wants to detect. For example, detecting a difference of 90% (λ= 1 and λ = 20) requires about 20 traps, but detecting a difference of 30% between λ = 14 and λ = 20, requires more than 90 traps. We used simulations to estimate the sample sizes (see Kéry and Royle [2]), which were not included because the manuscript was already large. Finally, also consider that the precision of the abundance estimation can be increased (thus allowing the detection of smaller differences) not only by increasing the density of traps, but also by repeated sampling, which we did.

4. I have reviewed and edited other articles using this technology and the companies unwillingness to provide methodological details is always frustrating, but beyond the authors control.

We share the editor’s frustrations.

5. Reviewer #2 clearly had some important insights, but the interaction became contentious and unproductive. I do think the manuscript improved in in response Reviewer #2s comments.

We agree that the manuscript improved in response to Rev 2 comments.

6. Other optional suggestions from the editor beyond addressing Reviewer #3' queries.
   a. I would recommend that in addition to that, that the authors attempt to reduce the results and discussions sections by at least 50%.

The result and discussion have been reduced by 100 lines.

b. I would focus on what was observed, specifically for Aedes aegypti and at least a paragraph on if cities in the US should consider using this technology

We, the authors, would require more experimental information before making a recommendation to US cities considering using this technology; there are still questions about how to best implement it in combination with other control strategies.

c. Consider eliminating the discussion of the impact of rainfall and temperature. As you point out, the issues could not be addressed statistically. You might indicate how large a study would be required to do so adequately and stop there, with one sentence indicating that clearly these factors helped drive some of the temporal variation you saw.

We have removed the discussion on weather. We now provided a regression table demonstrating that treatment had a significant effect on the mean number of females.

d. The discussion of the larval habitats for aegypti versus albopictus, was interesting, but a better designed study would be needed to address this question.

Agreed, we have removed that section.

e. A brief discussion of the merits of this statistical approach over others

We have reduced the size of the discussion

f. You did a good job discussion the design limitations but provide a concise summary and let it rest.

Thank you, see above.

g. I make those suggestions, because I felt the manuscript rambled endlessly and obscured a clear and important finding, 95% reduction in adult female aegypti densities.

We have reduced the size of the manuscript. Removed the comparison between the Negative Binomial and the N-mixture model; removed the comparison to Oxitech’s Brazil experiments; moved the overdispersion index to the supplemental section; removed the weather discussion.

h. You might consider moving more of the text to supplementary information and keeping the main body of the manuscript much simpler.

See above.

Again, the above paragraph are suggestions.

Amy

Below are the reviewer comments.

Reviewer's Responses to Questions

Key Review Criteria Required for Acceptance?

As you describe the new analyses required for acceptance, please consider the following:

# Methods

Are the objectives of the study clearly articulated with a clear testable hypothesis stated? Is the study design appropriate to address the stated objectives? Is the population clearly described and appropriate for the hypothesis being tested? Is the sample size sufficient to ensure adequate power to address the hypothesis being tested? Were correct statistical analysis used to support conclusions? Are there concerns about ethical or regulatory requirements being met?

## Reviewer #2:

(No Response)

## Reviewer #3:

I believe the methods for determining whether the Ae. aegypti population decreased were adequate. The additional analyses conducted in response to Reviewer #3 could be placed in the Supplement as sensitivity analyses. With the most recent changes, validation of the N-mixture method is now also adequate. However, concerns remain regarding the Ae. albopictus analysis. See comments on Results.

See below.

# Results

Does the analysis presented match the analysis plan? Are the results clearly and completely presented? Are the figures (Tables, Images) of sufficient quality for clarity?

## Reviewer #2:

(No Response)

## Reviewer #3:

Though not intuitively organized, as pointed out by Reviewer #2, the Results are largely sound and most issues have been resolved.

Starting with the statement on line 324, "Interestingly, the week-to-week variation appeared to be related to rainfall events (Fig. 1)," and continuing through the rest of the section to line 350, the authors spend 400 words describing rainfall and temperature fluctuations that may have influenced Ae. albopictus levels instead of the intervention. The authors are most frank in their responses to reviewers about the limitations of the Ae. albopictus analyses. They seem to feel that because they have not attached statistical language to these findings and have occasionally inserted words such as "probably" in front of their suppositions that the inclusion of these "findings" is legitimate. Reviewer #2 explains biological mechanisms that would make it unlikely that some of the relationships the authors claim as findings actually explain the data observed, yet these comments have not been adequately addressed. More fundamentally, I simply do not view these as "results." The authors' rationale that they cannot perform any statistical analysis to associate the weather patterns with the Ae. albopictus levels because of the low number of weeks sampled does not open the door for them to assert whatever they want -- a range of relationships in precipitation and temperature levels, with varying number of weeks between the weather event and the supposed responding mosquito bloom.

Additionally, the spatial aggregation results do not figure into the Discussion and Conclusions. As such, I believe they could be removed or relegated to the Supplement, reducing the length and complexity of the paper.

We have removed the section starting with line 324 and we now provide the regression table for treatment and time as effects for the mean number of females for both species. However, to accomplish this, we had to remove the dispersion index “layer” from the model because the MCMCs failed to converge after 1 week of search time.

After removing the dispersion layer, and adding the linear model, the effect of the treatment on Ae albopictus became noticeable, the expected mean number of females increased in the treated area, but the credibility intervals also increase (there is less precision). We can see that treatment affected the abundance of Ae albopictus, the effect was statistically less than one but greater than zero. However, without previous data it is unclear what the carrying capacity of the area for Ae albopictus was. Meaning, would the increase be greater if carrying capacity was larger? Or, after removing Ae aegypti the abundance of Ae albopictus only increases less than 100% because the species overlap is not large. The carrying capacity can be infer from the yearly dynamics of the population when it reaches its plateau.

We provide the dispersion index estimation on its own as supplemental material.

# Conclusions

Are the conclusions supported by the data presented? Are the limitations of analysis clearly described? Do the authors discuss how these data can be helpful to advance our understanding of the topic under study? Is public health relevance addressed?

## Reviewer #2:

(No Response)

## Reviewer #3:

In line with the comments on the Results, I think the conclusions relating to weather in the Discussion need to be significantly scaled back and be shaded with substantially more caution. The statement added about skepticism regarding the possibility of the intervention increasing Ae. albopictus levels goes in the wrong direction -- there is some suggestion in this data that this occurred, and nothing the authors have presented provides a compelling reason to disregard that signal. The small number of weeks presented is not a reason to disregard it when there could be harm done by ignoring it.

We added a linear model, to both species, with treatment and time as effects of the mean number of females. The signal became clearer, there were more Ae albopictus in the treatment area than in the untreated area, and treatment statistically explains the difference. However, we still have questions about the size of the effect treatment had on Ae albopictus, see above. We tracked Ae albopictus because we wanted to know if removing Ae aegypti would increase Ae albopictus. However, we still think this observation should be repeated using a larger period ( ) and a better metric like a community model. Regarding the length, we tried to say that we likely missed the expansion phase of the population when talked about the number of weeks.

--------------------

# Editorial and Data Presentation Modifications?

Use this section for editorial suggestions as well as relatively minor modifications of existing data that would enhance clarity. If the only modifications needed are minor and/or editorial, you may wish to recommend “Minor Revision” or “Accept”.

## Reviewer #2:

(No Response)

## Reviewer #3:

(No Response)

# Summary and General Comments

Use this section to provide overall comments, discuss strengths/weaknesses of the study, novelty, significance, general execution and scholarship. You may also include additional comments for the author, including concerns about dual publication, research ethics, or publication ethics. If requesting major revision, please articulate the new experiments that are needed.

## Reviewer #2:

(No Response)

## Reviewer #3:

Several comments from the original reviews were not addressed, indicated as addressed but not actually addressed, or answered in the response to the reviewers but not addressed in the manuscript. The original comments should be re-reviewed to ensure all have been appropriately addressed. In general, if a reviewer is confused about something, it should be clarified. Particularly, those reviewers that appeared to be subject matter experts should be considered as the target audience, and the paper clarified such that it would be understandable to them without additional explanation.

We went over the previous submitted version of the manuscript as well as the reviewers' responses, and we think that all comments were addressed. To handle a missing response(s), we would need a more concise example(s).

# Cited Literature

1.	Wasserstein RL, Lazar NA. The ASA's Statement on p-Values: Context, Process, and Purpose. The American Statistician. 2016;70(2):129-33. doi: 10.1080/00031305.2016.1154108

10.1080/00031305.2016.1154108</p>.

2.	Kéry M, Royle JA. Applied Hierarchical Modeling in Ecology: Analysis of distribution, abundance and species richness in R and BUGS. 1st ed. London, UK: Academic Press; 2015 2016.
